# Impact of Diverse Immune Evasion Mechanisms of Cancer Cells on T Cells Engaged by EpCAM/CD3-Bispecific Antibody Construct AMG 110

**DOI:** 10.1371/journal.pone.0141669

**Published:** 2015-10-28

**Authors:** Wibke Deisting, Tobias Raum, Peter Kufer, Patrick A. Baeuerle, Markus Münz

**Affiliations:** 1 Amgen Research (Munich) GmbH, Munich, Germany; 2 MPM Capital, Cambridge, Massachusetts, United States of America; Université Paris Descartes, FRANCE

## Abstract

**Background:**

Bispecific T cell engager (BiTE^®^) are single-chain bispecific antibody constructs with dual specificity for CD3 on T cells and a surface antigen on target cells. They can elicit a polyclonal cytotoxic T cell response that is not restricted by T cell receptor (TCR) specificity, and surface expression of MHC class I/peptide antigen complexes. Using human EpCAM/CD3-bispecific BiTE^®^ antibody construct AMG 110, we here assessed to what extent surface expression of PD-L1, cytoplasmic expression of indoleamine-2,3-deoxygenase type 1, Bcl-2 and serpin PI-9, and the presence of transforming growth factor beta (TGF-β), interleukin-10 (IL-10) and adenosine in culture medium can impact redirected lysis by AMG 110-engaged T cells.

**Methods:**

The seven factors, which are all involved in inhibiting T cell functions by cancer cells, were tested with human EpCAM-expressing Chinese hamster ovary (CHO) target cells at levels that in most cases exceeded those observed in a number of human cancer cell lines. Co-culture experiments were used to determine the impact of the evasion mechanisms on EC_50_ values and amplitude of redirected lysis by AMG 110, and on BiTE^®^-induced proliferation of previously resting human peripheral T cells.

**Findings:**

An inhibitory effect on redirected lysis by AMG 110-engaged T cells was seen upon overexpression of serpin PI-9, Bcl-2, TGF-βand PD-L1. An inhibitory effect on induction of T cell proliferation was only seen with CHO cells overexpressing IDO. In no case, a single evasion mechanism rendered target cells completely resistant to BiTE^®^-induced lysis, and even various combinations could not.

**Conclusions:**

Our data suggest that diverse mechanisms employed by cancer cells to fend off T cells cannot inactivate AMG 110-engaged T cells, and that inhibitory effects observed *in vitro* may be overcome by increased concentrations of the BiTE^®^ antibody construct.

## Introduction

Therapies engaging a patient’s cytotoxic T cell response have proven to effectively treat and eventually cure cancer. For instance, adoptive transfer of ex-vivo expanded tumor-resident T cells [1], inhibition of immune escape by the PD-1/PD-L1 axis by monoclonal antibodies (mAbs) [2], intra-lesional injection of an oncolytic virus [3], or enhancing T cell differentiation and depleting regulatory T cells by CTLA-4-antagonistic mAbs [4] have all shown partial and complete responses in late-stage melanoma, a positive impact on progression-free or overall survival, and long-term remission if not cure in a small proportion of patients. Currently, response rates of these approaches are limited, which is why extensive biomarker programs aim at understanding resistance and multiple clinical programs search for combinations potentially increasing response rates and long-term benefit. All of the above approaches enable the generation, expansion and systemic spread of tumor-specific T cell clones that recognize cancer cells by their specific MHC class I/peptide complexes.

BiTE^®^ antibody constructs engage cytotoxic T cells by a fundamentally different mechanism [5]. They use a soluble adapter to connect a surface target antigen on cancer cells—as is typically recognized by monoclonal antibody therapies—with the invariant CD3ε subunit of any T cell receptor (TCR) on T cells. Potentially all pre-existing cytotoxic T cells in a patient can be engaged by this approach, of which effector memory T cells seem to make the dominant contribution to anti-tumor activity [6]. With the BiTE^®^ technology, T cell recognition and activation is no longer dependent on T cell clones bearing a specific TCR, not on transport and expression of MHC I molecules to the cancer cell surface, or on the proteolytic generation, transport and surface display of peptide antigens [5]. The CD19/CD3-bispecific blinatumomab has provided clinical proof-of-concept that this non-natural engagement of T cells is highly effective and can elicit in a large proportion of ALL and NHL patients meaningful clinical responses [7–9]. Blinatumomab (Blincyto^™^) was recently approved by the FDA for treatment of patients with Philadelphia chromosome-negative relapsed/refractory B cell precursor ALL.

Here, we used AMG 110, a well characterized EpCAM/CD3-bispecific BiTE^®^ antibody construct that is clinically being tested in late-stage cancer patients with different carcinomas [10, 11].

Cancer cells can be selected during tumor progression for numerous immune evasion mechanisms, which for instance can impact MHC class I/peptide presentation [12, 13], or the generation, differentiation, survival, migration and expansion of specific cytotoxic T cell clones. In the present study, we investigated to what extent seven frequent evasion mechanisms impact the BiTE^®^ mode of action, which can potentially engage any pre-existing cytotoxic T cell in patients. We therefore focused on those mechanisms that can potentially impact cytotoxic T cell performance and left out those that, for instance, impair specific T cell recognition by MHC I/peptide complexes. To this end, we established rodent CHO cell lines expressing human EpCAM as surface target antigen that either overexpress proteins known to have immune evasion potential or where the immunosuppressive factor was added to the culture medium. Potency and amplitude of redirected CHO-EpCAM target cell lysis and induction of T cell proliferation in response to AMG 110 were used as readouts for cytotoxic T cell performance.

With PD-L1, adenosine, IL-10 and TGF-β we explored factors frequently produced by cancer cells that bind negative regulatory surface receptors expressed on cytotoxic T cells. PD-L1 is expressed on the surface of tumor cells and prevention of binding its inhibitory receptor PD-1 on T cells by mAb monotherapies was shown to have pronounced anti-tumor activity in clinical trials [14, 15]. Adenosine can be synthesized by cancer cells and binds the A2a and A3 adenosine receptors on T cells, which can inhibit effector functions [16, 17]. IL-10 is considered an anti-inflammatory cytokine with broad function. Because cancer cells can secrete IL-10, a role in the escape of immunosurveillance has been ascribed to IL-10 in tumors [18]. By transcriptional repression, TGF-β secreted by cancer cells can broadly downmodulate effector T cell functions, for instance, by reducing expression of granzymes A and B, perforin and interferon-gamma (IFNγ) upon receptor binding [19]. The cytoplasmic protease inhibitor serpin PI-9 can directly block the enzymatic activity of granzyme B that is eventually delivered into the cytosol of cancer cells through a cytolytic synapse as formed by T cells [20]. With Bcl-2, we studied an anti-apoptotic cytosolic protein that can decrease the susceptibility of cancer cells to induction of apoptosis by granzyme B [21]. With IDO, we explored a catabolic enzyme that when overexpressed by cancer cells can starve the microenvironment of the essential amino acid tryptophane and release immunosuppressive metabolites, like l-kynurenine or 3-hydroxykynurenine, which can tolerize T cells [22, 23].

With the exception of IFNγ-induced IDO, all other evasion factors were studied at levels in transfected CHO cells that by far exceeded those found in six human cancer cell lines. Despite studying obviously high levels of evasion factors, we have observed only modest effects of some on redirected lysis and induction of T cell proliferation. In most cases, inhibitory effects could be compensated by higher dosing of BiTE^®^ antibody construct AMG 110, or be reversed by pharmacological means. The BiTE^®^-engaged T cells may have the potential to be active against cancer cells expressing a variety of frequent immune evasion mechanisms.

## Material and Methods

### Reagents

AMG 110 was provided by Amgen Research (Munich) GmbH. It was produced by recombinant DNA technology and purified via a C-terminal hexa-histidine tag as previously described [24].

### Antibodies

The following antibodies were applied. For flow cytometry: mouse anti-human PD-L1-APC (M1H1, eBiosciences), mouse anti-human CD3-FITC (UCHT1; BD Biosciences), mouse anti-human CD25-APC (M-A251; BD Biosciences), mouse anti-human EpCAM (B302/323/A3; Abcam), goat anti-Mouse IgG (H+L)-APC (Jackson Immuno Research), mouse anti-human TGF-β-PE (9016; R&D Systems); for intracellular FACS or Western blot analysis: mouse anti-human PI-9 (7D8; Abcam), mouse anti-human GrB-APC (GB11; BD Biosciences), mouse anti-human Bcl-2 (100/D5; Thermo Scientific), mouse anti-human β-Actin (AC-15; Thermo Scientific), rabbit anti-human IDO (Abcam), goat anti-Mouse and anti-Rabbit HRP-coupled detection antibodies (Thermo Scientific); stimulating and neutralizing antibodies: mouse anti-human CD3 (OKT-3; Janssen-Cilag), mouse anti-human CD28 (L293; BD Biosciences), mouse anti-human TGF-β (1D11; R&D Systems) and mouse anti-human PD-L1 (M1H1; eBiosciences)

### Construction, cultivation and testing of escape protein and of stably human EpCAM-expressing CHO cell lines

Parental chinese hamster ovary (CHO) cells expressing full-length human EpCAM under the control of the EF1α promotor (provided by Amgen Research (Munich) GmbH) were stably transfected with a second expression plasmid coding for either human PI-9, IDO, Bcl-2, PD-L1, TGF-β1, IL-10 or the corresponding mock vector. Except for Bcl-2 (generated by gene synthesis; Geneart), all escape proteins were cloned from human cancer cell lines.

Subsequent selection and amplification of protein expression were achieved by addition of methotrexate (Sigma-Aldrich), L-alanosine (TRC, Toronto) and dcF (Hospira). CHO EpCAM: Escape protein double-transfectants were grown in suspension in HyQ medium (HyClone) supplemented with 1% penicillin/streptomycin (Biochrom AG), 500 nM methotrexate, 10 mM HEPES (Biochrom AG), 1.1 mM adenosine (Sigma-Aldrich), 50 μM L-alanosine, 1 mM uridine (Sigma-Aldrich) and 0.1–1 μM dcF at 37°C in 5% CO_2_ humidified incubator.

Expression of membrane-bound proteins EpCAM and PD-L1 and membrane-bound TGF-β was analyzed by flow cytometry, expression of PI-9 by intracellular FACS or Western blot analysis. For comparison of expression levels, the relative median (= median sample/median control) of the samples were calculated. Expression of intracellular IDO and Bcl-2 was determined by Western blot analysis and quantified with Image J software. Expression levels of secreted IL-10 and TGF-β proteins were assessed in cell supernatant of 2.5x 10^5^/ml after 48 h of culturing by ELISA using Quanti Glo IL-10 Elisa Kit (R&D Systems) and a Tecan SpectraFluor Plus (MTX Lab Systems) for detection or TGF beta Elisa Kit (Abcam) and an ELISA reader Power Wave X (BioTec Instruments) for detection.

For functional analysis of TGF-β secreted from CHO transfectants, cell culture supernatant from 2.5x 10^5^/ ml was collected after 48 h. One x10^6^ CD3^+^ T cells were stimulated for 96 h with plate-bound CD3/CD28/IL-2 in the supernatant mixed with fresh medium (25% medium: 75% supernatant). Effects of TGF-β were analyzed by determining the expression of GrB by intracellular FACS analysis. The same assay was used for functional analysis of recombinant TGF-β and TGF-β neutralizing antibody. For functional analysis of adenosine effects, T cells were stimulated by plate-bound CD3/CD28/IL-2 with and without 1 mM adenosine. Effects of adenosine were determined by measuring CD25 expression by FACS analysis after 24 or 96 h.

### Human tumor cell lines and cell culture

A549 [25], BxPC3 [26], KATOIII [27], SKBR3 [28] cells were all grown in RPMI1640 medium (Biochrom AG) supplemented with 10% fetal calf serum (Biochrom AG), 1% penicillin/streptomycin (Biochrom AG), 50 μM 2-mercaptoethanol (Gibco), 1% nonessential amino acids (Biochrom AG), 1 mM sodium pyruvate (Biochrom AG) and 1 mM HEPES (Biochrom AG). SW480 [29] cells were grown in RPMI1640 medium supplemented with 10% fetal calf serum and 1% penicillin/streptomycin. A431 [25] cells were grown in DMEM (Biochrom AG) with 10% fetal calf serum and 1% penicillin/streptomycin. All cell lines were cultured according to standardized cell culture procedures. For induction of IDO and PD-L1 proteins, cells were treated for 48 h with 1000 U/ml IFNγ (Sigma-Aldrich).

### T-cell isolation, stimulation and in vitro assays

Human blood was donated by healthy volunteers, who were medical illuminated and registered in an internal blood bank of Amgen Research (Munich) GmbH. Each donor was assigned a number that was used for naming the blood samples. The blood was obtained directly before usage by a trained team not including the authors of this paper. In some cases the blood drawn was separated between different studies. Before each donation, volunteers provided a written consent for their participation in medical research. To protect the health of the donors, the blood pressure was measured and donors were asked about their current state of health before taking the blood. Moreover, blood values of each volunteer were checked quarterly by an external laboratory and strict intervals between two donations were kept. Each donor only supplied a small volume of blood not exceeding the range of blood samples drawn for minor investigations during preventive medical checkups. Therefore, no permission of an ethic committee was sought. PBMCs were isolated from heparinized blood by Ficoll (Biochrom) density gradient centrifugation using standard procedures. For elimination of residual erythrocytes, cells were incubated with erythrocyte lysis buffer (155 mM NH_4_Cl, 10 mM KHCO_3_, 0.1 mM Na_2_EDTA) for 5 min at 4°C. PBS Dulbecco (Biochrom) with 2% FCS was added to stop the reaction. After a centrifugation for 5 min at 600 x g, the supernatant with lyzed erythrocytes was discarded. CD3^+^ T cells were obtained by MACS magnetic bead cell separation using the pan T cell Isolation kit II (Miltenyi). CD8^+^ T cells were separated from freshly isolated or pre-stimulated PBMCs by either depletion of CD4^+^ and CD56^+^ cells using mouse anti-human CD4 (L200; BD, Heidelberg) and mouse anti-human CD56 antibodies (B159; BD), sheep anti-mouse IgG beads (Invitrogen) and Dynal magnets, or by using the MACS CD8^+^ Isolation Kit (Miltenyi). T cells were stimulated with plate-bound anti-CD3 (OKT-3; Janssen-Cilag) and anti-CD28 (BD) antibodies and 20 U/ml recombinant human interleukin-2 (IL-2, Chiron Corperation Ltd) in RPMI1640 medium (Biochrom AG), supplemented with the same ingredients as listed above. Supplemented RPMI was used for cultivation of T cell and all *in vitro* assays. Further additions as adenosine (Sigma-Aldrich), tryptophane (Sigma-Aldrich) recombinant cytokines (Miltenyi), or neutralizing antibodies were added directly into the culture medium at the beginning of the incubation period.

### FACS-based cytotoxicity assays

Target cells were labeled with Vybrant DIO or DID (Life Technologies) fluorescent membrane dye, adjusted to 1.25x 10^5^ cells/ml and co-incubated in 96-well plates with freshly isolated CD3^+^ T effector cells at an effector-to-target (E:T) ratio of 4:1 or with pre-stimulated CD8^+^ T effector cells at an E:T ratio of 1:1 in the presence of AMG 110 serial dilution. Further additions were added directly to the assay medium. Incubation was conducted at 37°C, in a 5% CO_2_ humidified atmosphere. After 24–72 h, living and dead cells were collected, resuspended in PBS/2% FCS with 1 μg/ml propidium iodide (PI) (Sigma-Aldrich), and analyzed in a FACS Canto^™^ II flow cytometer (Becton Dickinson) equipped with FACSDiva software. Cytotoxicity was determined in duplicates by quantifying the labeled and PI positive non-viable target cells and the labeled PI negative target cells. Percentage of lysis was analyzed with respect to BiTE^®^ concentration using Prism 5 software (GraphPad). Analysis with Prism 5 included creation of sigmoidal dose-response curves, calculation of EC_50_ values as well as determination of top and bottom percentage of BiTE^®^-dependent lysis for calculation of lysis amplitude for a given time point. In order to compare the impact of escape proteins on BiTE^®^-induced redirected lysis relative change in EC_50_ was calculated by the formula: (EC_50_ values of escape transfectants)/(EC_50_ values of parental control cells) and relative change in amplitude values by (amplitude of Escape transfectants)/(amplitude of parental control cells). Each assay was at least performed in duplicates.

### CFSE-based proliferation assays

To assess the proliferative potential of T cells in the presence of immune escape proteins, T cells were labeled with carboxyfluoresceine diacetate succinimidyl esther (CFSE) according to the manufacturer`s instructions (Molecular Probes). Labeled T cells were co-incubated with CHO cell lines stably expressing EpCAM and one of the evasion proteins or control cells +/- adenosine in an incubator with 37°C, 5% CO_2_ atmosphere for 120 h in 48-well flat bottom plates (Nunc) in the presence or absence of 1 μg/ml AMG 110. The number of T cells was kept constant at 1x 10^4^. CHO cells were washed twice to remove selection medium and were adjusted to E:T ratios of 1:8, 1:1 and 4:1. After the incubation period, proliferation of CD3^+^ T cells was determined by monitoring CFSE distribution by flow cytometry using a FACS Canto^™^ II flow cytometer and FACS DIVA^™^ software. Subsequent analysis was performed using FlowJo 7.6.5 software. All assays were performed at least in duplicates.

### Statistical analysis

Statistical analysis of EC_50_ values and amplitudes significant outliers were excluded with the Grubb′s test. Afterwards p values were calculated with Prism 5 (GraphPad Software) using unpaired t tests with Welch′s correction.

## Results

### Transfected CHO cell lines express high levels of human immune evasion factors

Stably transfected, human EpCAM-expressing CHO cells were characterized for expression levels of human immune evasion proteins in comparison to natural levels found in six human cancer cell lines A549 (lung), BxPC3 (prostate), KATOIII (gastric), SKBR3 (breast), SW480 (colorectal), and A431 (skin). For IDO and PD-L1, cancer cells were in addition stimulated for 48 h with 1,000 U/ml IFNγ, which is known to induce the proteins [30, 31]. Various methods were employed to quantify and compare expression levels, including FACS (for serpin PI-9, PD-L1, EpCAM and TGF-β), ELISA (for TGF-β and IL-10) and Western blotting (for Bcl-2 and IDO; see analyses in S1 Fig). Western blot analyses confirmed the correct molecular size and the use of specific detection antibodies the identity of the respective proteins in cancer cell lines and transfected CHO cells. A summary of expression data shows that—apart from IDO after stimulation of A431, A549, BxPC3 and KATOIII cells with IFNγ—in all cases evasion proteins were expressed in stably transfected CHO cells to a much higher level than in the six cancer cell lines. The expression of the target antigen EpCAM in most cell lines (A431, BxPC3 and KATOIII, SkBR3 and SW480) was in the range of CHO transfectants (Factor 2–9 fold less) whereas A549 showed only moderate EpCAM expression (50x less than transfected CHO cells) (Table 1).

**Table 1:** Evasion protein expression in human cancer cell lines and stably expressing CHO transfectants.

Rel. Expression Level [%]	CHO transfectants	A431	A549	BxPC3	KATOIII	SKBR3	SW480
**PI-9**	100	9.5	20.1	11.1	7.1	18.5	13.2
**Bcl-2**	100	1	0	1.8	0.2	0.8	0
**IDO**	100	0	0	0	0	0	0
**IDO + IFNγ**	100	124.2	413.5	745.7	166.6	n.d.^a^	0
**IL-10**	100	0.9	0.7	0.3	1.2	0	18.7
**TGF-β**	100	0.03	0.2	0.03	0	0.06	0.2
**PD-L1**	100	2.4	0.08	0.4	0.08	0.3	0.08
**PD-L1 + IFNγ**	100	2.5	0.6	1.3	0.3	0.1	0.08
**EpCAM**	100	10.7	1.9	11.1	45.5	20.4	35.0

PI-9, PD-L1 and EpCAM expression was determined by FACS analysis, Bcl-2 and IDO expression was assessed by Western blot and subsequent analysis with Image J software, and TGF-β and IL-10 were detected with specific ELISA assays. The expression level observed in the CHO EpCAM transfectants was set to 100%. Stimulation of cells with IFNγ was at 1000 U/ml for 48 h.

^a^n.d. = not determined.

### IDO showed pronounced inhibitory effect on AMG 110-induced T cell proliferation

**Fig 1:**
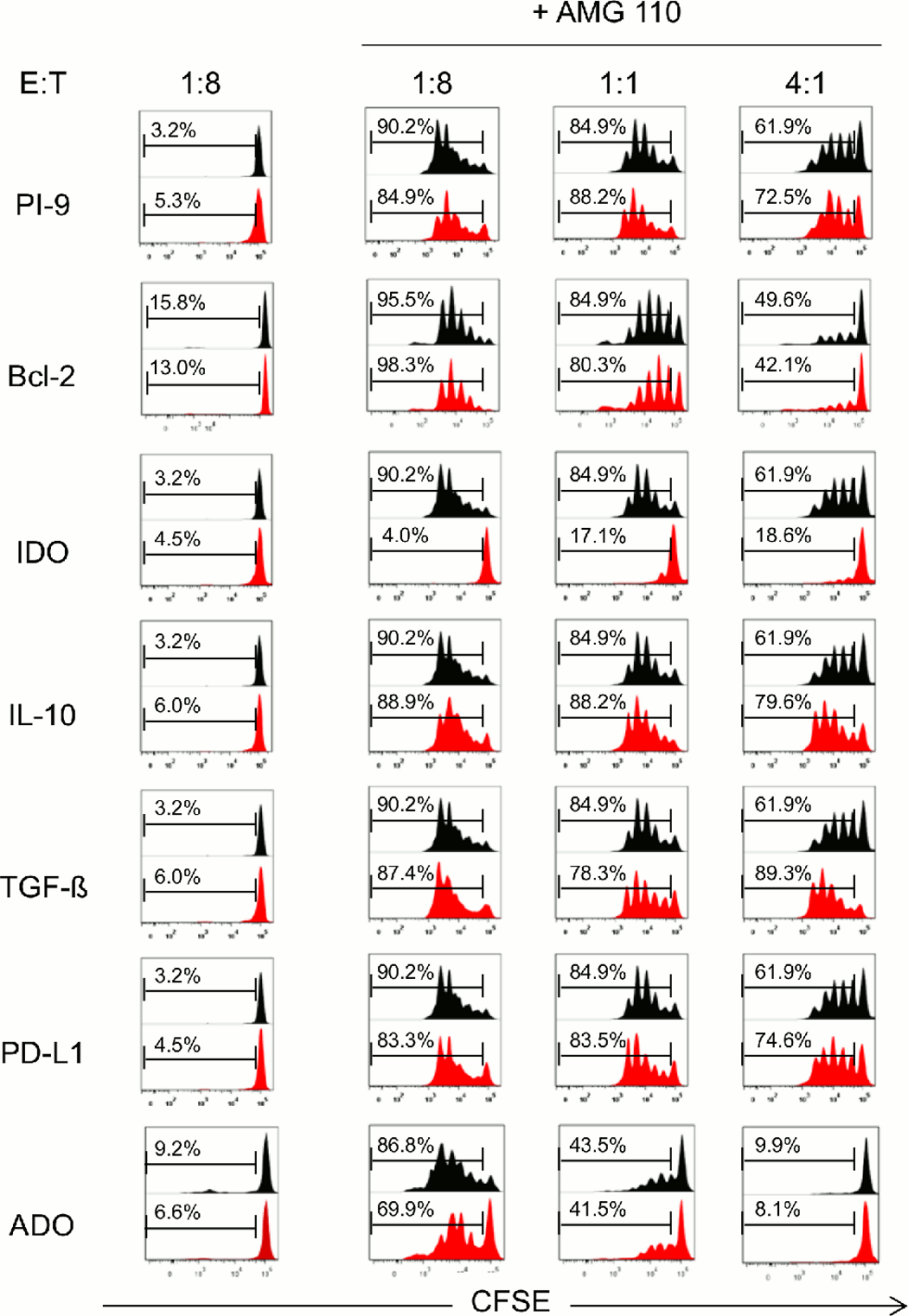
Impact of diverse immune evasion mechanisms on AMG 110-mediated T cell proliferation. Human CD3^+^ T cells were labeled with CFSE and co-cultured at effector to target (E:T) ratios of 1:8, 1:1 and 4:1 in 48-well plates with CHO cell lines expressing human EpCAM and one of the six stably transfected immune evasion proteins (PI-9, Bcl-2, IDO, IL-10, TGF-β or PD-L1) (red), or with parental EpCAM^+^ CHO cells in the presence of 1 mM adenosine (ADO) in the culture medium (black: parental cells). CFSE signals in cells were analyzed by flow cytometry. For each evasion protein one representative experiment is shown. Co-culture in the absence of AMG 110 did not gave CSFE signals, whereas co-culture in the presence of 1 μg/ml AMG 110 showed cycles of cell division affected by E:T ratio. Three different human PBMC were used.

AMG 110 and other BiTE^®^ antibody constructs have the capability to potently activate T cells, but only in the presence of target cells [32]. We here investigated the impact of seven immune escape factors on initiation of T cell proliferation by AMG 110 at three different effector to target (E:T) ratios after co-culture for 120 h. In the absence of AMG 110, none of the transfected or control CHO cell lines elicited a proliferation of the CD3^+^ T cell population within added peripheral mononuclear cells (PBMCs) as monitored by FACS using CSFE staining (Fig 1). In the presence of 1 μg/ml AMG 110, control CHO-EpCAM cells (black) as well as CHO cells with evasion factors (red) showed strong T cell proliferation signals indicative of multiple rounds of cell cycling (Fig 1). Somewhat stronger signals were observed with increasing E:T cell ratios (compare E:T ratio of 1:8 with 4:1). A significant difference between control and evasion factor was only noted for cells expressing IDO. At all three E:T ratios, AMG 110 could barely induce T cell proliferation in CHO-EpCAM cells stably transfected with human IDO cDNA. A missing impact of TGF-β and IL-10 was confirmed by addition of recombinant factors to the culture medium (see S2A Fig). For the experiments, three different human PBMC donors were used. one of them showed a weaker AMG 110 dependent proliferation (see Adenosine E.T = 1:1 and 4:1).

### Serpin PI-9, TGF-β, Bcl-2 and PD-L1 reduce the potency of redirected target lysis by AMG 110

**Fig 2:**
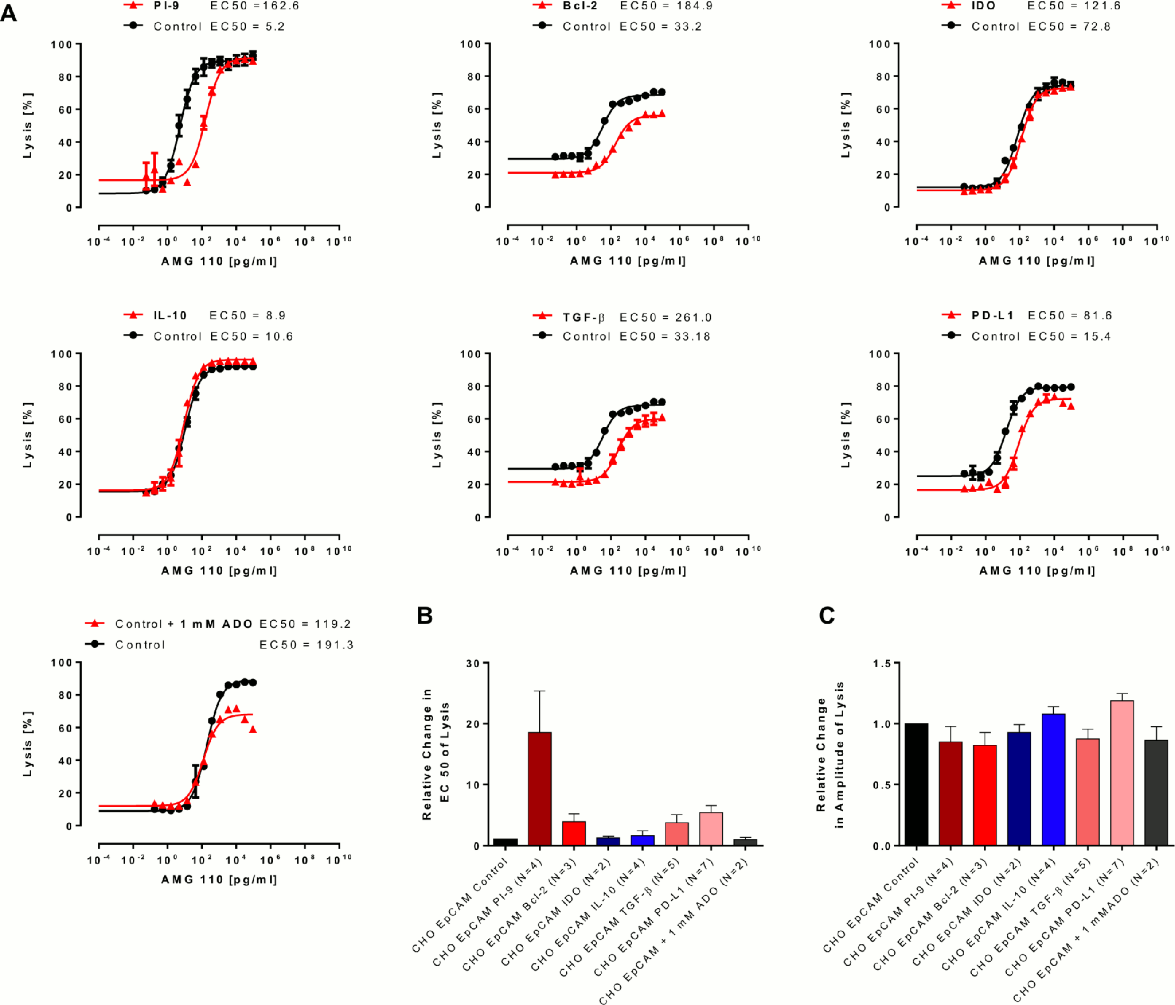
Impact of diverse immune evasion mechanisms on AMG 110-mediated redirected lysis of target cell. AMG 110 dose-dependent lysis was compared between parental EpCAM^+^ CHO cells and EpCAM^+^ CHO cells stably transfected with one of six human evasions proteins or in the presence of 1 mM adenosine. Human CD8^+^ T cells were used as effector cells at an E:T ratio of 1:1. Percentage of target cell lysis after co-culture for 24 h and EC_50_ values from sigmoidal dose-response response curves were determined in a FACS-based cytotoxicity assay. (A) Representative dose response curves are shown for parental EpCAM^+^ CHO cells (black) and EpCAM^+^ CHO cells expressing evasion proteins or incubated in the presence of 1 mM adenosine (red). (B) Mean EC_50_ values for redirected lysis were calculated from the indicated number of independent experiments. The relative change in EC_50_ values for lysis was determined by dividing the mean of EC_50_ values from evasion conditions by the mean of EC_50_ values from control conditions. Error bars represent SEM values. (C) Relative changes in the percentage of target cell lysis after 24 h at the plateau of dose response curves. The mean amplitude of lysis under evasion conditions was divided by the mean amplitude of lysis under control conditions. Error bars represent SEM values.

Using purified CD8^+^ T cells from healthy human PBMC donors, AMG 110 dose response analyses for redirected lysis compared EC_50_ values and the percentage of lysis between CHO-EpCAM control cells (black) and target cells in the presence of stably expressed or added evasion factors (red) (Fig 2). Co-culture was for 24 hours at an E:T ratio of 1:1. Depending on T cell donors, basal EC_50_ values for redirected lysis varied between 5 and 200 pg/ml AMG 110. The percentage of cell lysis ranged between 40 and 90%. Discrete shifts of dose response curves to higher EC50 values and lower percentages of lysis were noted for several evasion factors (Fig 2A). However, in no case, the presence of an evasion factor did abrogate redirected lysis. A quantitation of results is shown in Fig 2B and 2C.

The strongest inhibitory effect on the potency of redirected lysis was seen with the granzyme B inhibitor serpin PI-9 showing a mean 18-fold increase in EC_50_ values (Fig 2A and 2B), with a trend toward statistical significance (p = 0.07; N = 5). Second strongest was the effect of surface expression of PD-L1 on CHO-EpCAM cells with a >5-fold reduction in potency reaching statistical significance (p = 0.04; N = 7). Expression of TGF-β induced a 3-fold reduction in the potency of lysis with a strong trend (p = 0.06; N = 5). An inhibitory effect was confirmed by addition of recombinant TGF-β to the culture medium of CHO-EpCAM control cells (see S2B Fig). The >4-fold inhibitory effect of Bcl-2 on the EC_50_ value of lysis showed a weak trend (p = 0.09; N = 3), and a statistically significant albeit small reduction (see Fig 2C) in the percentage of lysed cells (p = 0.01; N = 4). None of the other effects on the efficacy of lysis or percentage of lysed cells described in Fig 2 reached statistical significance or showed a strong trend (S3A and S3B Fig). Similar results were obtained when CD3^+^ T cells were used instead of CD8^+^ T cells as effector cell population (see S4 Fig).

### The inhibitory activities of IDO, PD-L1, and TGF-β can be reversed by pharmacological means

**Fig 3:**
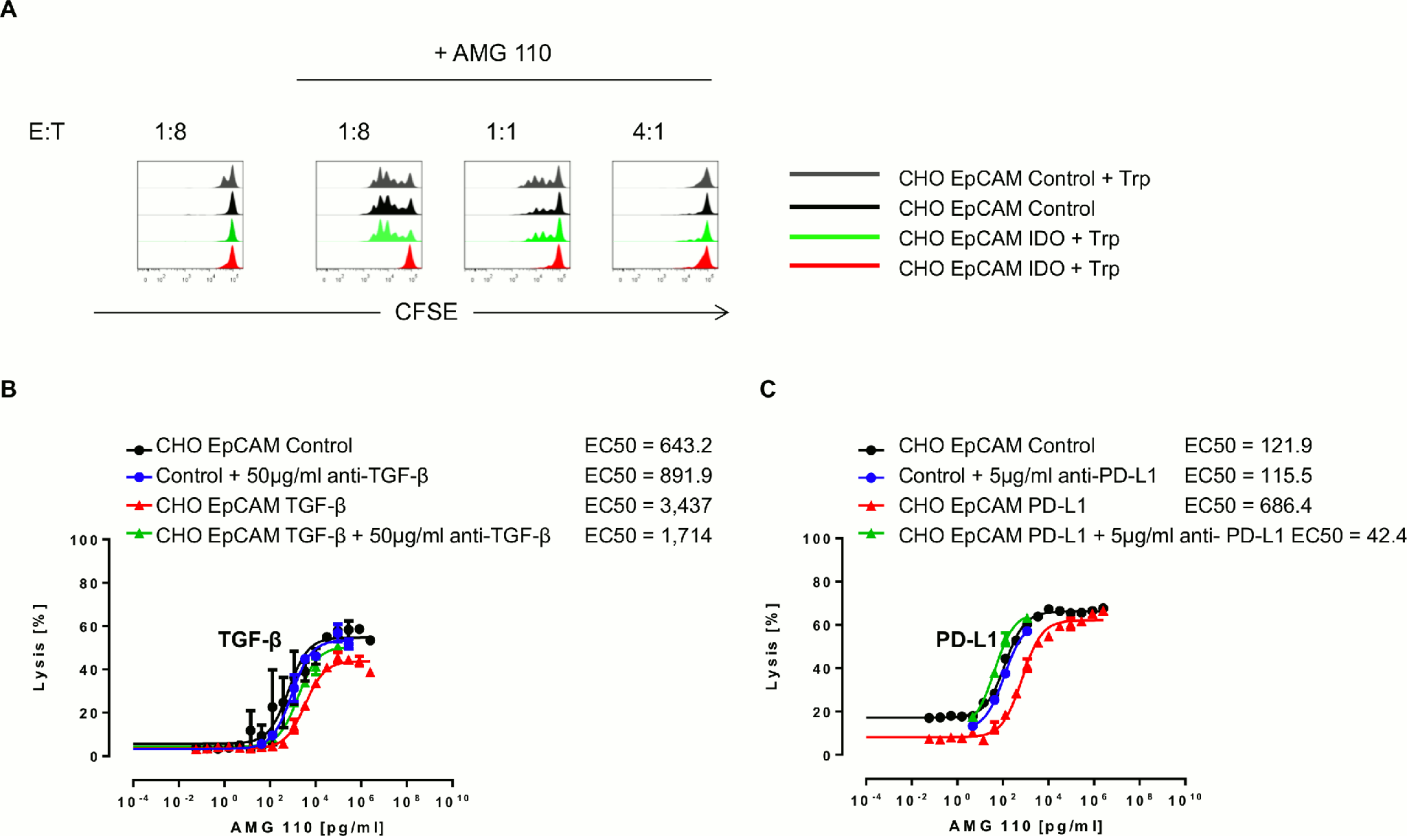
Reversal of inhibitory effects of PD-L1, IDO and TGF-β by pharmacological means. (A) Analysis of CD3^+^ T cell proliferation after 120 h of co-culture with control EpCAM^+^ CHO cells and IDO-expressing, EpCAM^+^ CHO cells in the presence or absence of 500 μM tryptophane (Trp) with and without 1 μg/ml AMG 110. (B) Dose-dependent, redirected target cell lysis of parental EpCAM^+^ CHO cells and EpCAM^+^ CHO TGF-β transfectants +/- 50 μg/ml TGF-β neutralizing anti-human TGF-β antibody in presence of AMG 110 and CD3^+^ T cells in an E:T ratio of 4:1 after 72 h incubation. (C) Dose-dependent redirected target cell lysis of control EpCAM^+^ CHO cells and EpCAM^+^ CHO PD-L1 transfectants with and without 5 μg/ml of an anti-human PD-L1-neutralizing antibody in the presence of AMG 110 and pre-stimulated CD8^+^ T cells. The E:T ratio was 1:1, the assay duration 24 h.

IDO was the only evasion factor in this study leading to strong inhibition of AMG 110-induced T cell proliferation (see Fig 1). To demonstrate that the inhibition was due to the enzymatic activity of IDO, we added 0.5 μM tryptophane to the cell culture medium and analyzed cell cycling by CFSE-based FACS. As shown in Fig 3A, replenishment with tryptophane completely reversed the inhibition of AMG 110-induced cell cycling in IDO-expressing CHO-EpCAM cells.

The inhibitory effect seen upon expression of TGF-β on redirected lysis of target cells expressing and secreting human TGF-β could be reversed in the presence of 50 μg/ml of a neutralizing anti-TGF-β antibody (Fig 3B). The decreased lysis of PD-L1 expressing CHO-EpCAM cells by AMG 110 could be fully reversed in the presence of 5 μg/ml of an antagonistic anti-PD-L1 antibody (Fig 3C). Anti-TGF-β and anti-PD-L1 antibodies or PI-9 inhibition by shRNA had no effect on lysis of cancer cell lines expressing the respective evasion factors at low levels (see S4D, S4E and S4F Fig).

### Redirected target cell lysis is observed even if all seven evasion mechanisms are present in co-culture

**Fig 4:**
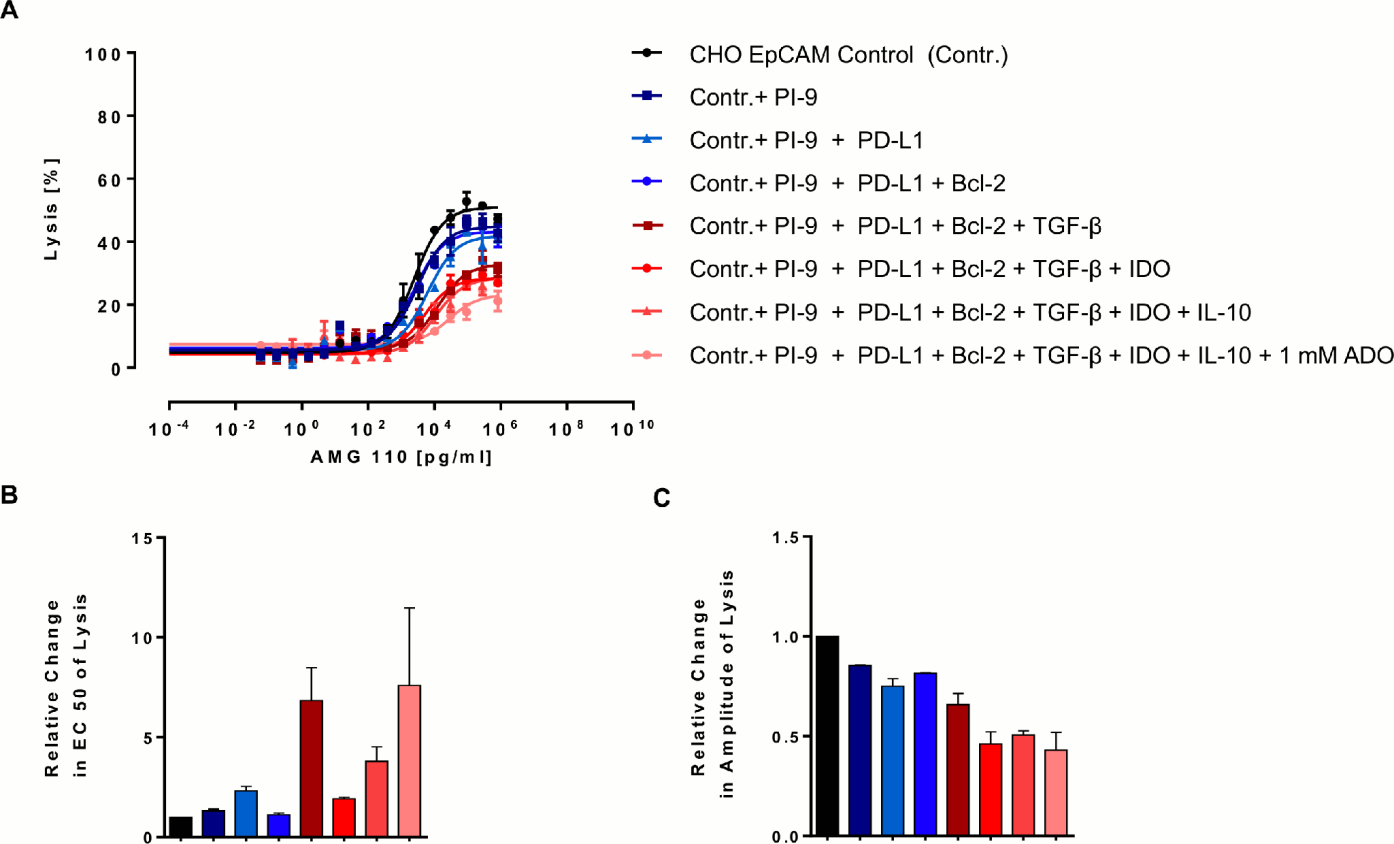
Combinatorial effect of evasion mechanisms on AMG 110-mediated redirected target cell lysis. Parental human EpCAM^+^ CHO cells and different combinations of EpCAM^+^ CHO cells expressing evasion proteins were co-cultured at an E:T ratio of 4:1 with CD3^+^ T cells and various AMG 110 concentrations for 72 h. For each curve an equal amount of control EpCAM^+^ CHO cells was replaced by an additional stable CHO cell line until the entire target cell population comprised evasion protein-expressing cells and contained 1 mM adenosine. (A) Target cell lysis was determined by a FACS-based cytotoxicity assays. Sigmoidal dose-response curves were created with GraphPad Prism software. Error bars represent SEM values. (B) Relative change in EC_50_ and (C) percentage of cell lysis after 72 h were calculated as described in Fig 3. Error bars represent SEM values from two independent experiments

Several evasion factors investigated in this study can act in *trans*, such as TGF-β, IL-10, PD-L1, IDO and adenosine. Only Bcl-2 and serpin PI-9 proteins, which are confined to the cytoplasm of cancer cells, cannot. We here attempted to create in co-culture an immunosuppressive environment by having all seven factors present at the same time. In one condition, each of the six transfected CHO-EpCAM cell lines was present at the same number and 1 mM adenosine added to the culture medium. Even under this condition, redirected lysis was still observed (Fig 4). However, a substantial impact on the percentage of lysis was evident with an increasing number of factors combined. The impact on the EC_50_ value of lysis showed a trend and did not exceed a 7-fold reduction.

## Discussion

This is the first study exploring the impact of diverse evasion mechanisms on the performance of cytotoxic T cells engaged by a BiTE^®^ antibody construct. Our experimental system used rodent target cells in co-culture with human T cells, which excludes mechanisms of regular T cell recognition based on matching MHC class I or co-stimulatory molecule interactions. T cell recognition was solely based on connecting human EpCAM on CHO-EpCAM cells with CD3ε on human T cells by the bispecific AMG 110 protein. Previous studies have shown that MHC I expression is dispensable for the activity of AMG 110 [33], and that T cell activation and ensuing target cell lysis is entirely dependent on proper recognition of the human EpCAM antigen [34]. By using stably transfected CHO-EpCAM cell clones, we established expression levels for six evasion proteins that, with the exception of IFNγ-induced IDO, exceeded by far those found in six human cancer lines. Our experimental system should therefore be suitable to faithfully study the isolated activity of an evasion factor on BiTE^®^-induced T cell proliferation and redirected lysis with a suitable background and acceptable signal strength.

Despite a high-level expression of evasion proteins or the abundance of soluble evasion factors in culture medium, we were surprised to observe only subtle changes in the performance of AMG 110-activated T cells. Most substantial was an inhibition of T cell cycling by IDO, and shifts in EC_50_ values of redirected lysis compared to control CHO-EpCAM cells in cells expressing serpin PI-9, PD-L1, TGF-β and Bcl-2, of which only the effect of PD-L1 reached statistical significance. The only statistically significant but small impact on the percentage of lysis was seen for Bcl-2. In all other cases, no significant inhibition was observed and higher concentrations of AMG 110 could typically compensate for the reduced percentage of lysis during the 24-72-h co-culture experiments.

What may explain the relative insensitivity of AMG 110-mediated T cell activation and lysis to most evasion factors? Firstly, our experimental system studying isolated factors may underestimate the impact of combinatorial effects of evasion factors. Heterogeneity of tumor cells has been comprehensively documented [35, 36] and may well extent to the number and combination of evasion factors selected during disease progression within tumor tissues. This is evident from the present study where more than one evasion protein was detectable in the six human cancer cell lines studied (see S1 Fig). In one experiment, we tried to address combinations of evasion factors by mixing all CHO cell clones in the presence of 1 mM adenosine. Even under this circumstance, redirected lysis was still detectable albeit at lower potency and reduced kinetics as reflected in a decrease of the amplitude of lysis. Another factor contributing to the insensitivity of AMG 110-engaged T cells could be the release of pro-inflammatory cytokines by BiTE^®^-activated T cells [32]. IFNγ, interleukins 2 and 6, TNF-α and many other immune-stimulatory cytokines were found to be released upon BiTE^®^ stimulation *in vitro*, in mouse studies and in patients [37, 38]. These could in part counteract the activity of evasion factors by induction of activating signal transduction pathways in T cells. For example, the addition of IL-2 has been shown to overcome PD-L1-dependent inhibition of proliferation [39].

Apart from that, the *in-vitro* assays investigating redirected lysis were executed with an E:T ratio of 1:1. In vivo, the number of T cells in the tumor might be less favorable. The ability of BiTE^®^ antibodies to engage all existing effector T cells in the body to eliminate mulitiple target cells might decrease the effects of inhibition of T cell proliferation (like IDO). Nevertheless, a surplus of tumor cells might require local t cell expansion.

Another possibility is that evasion factors need to act for prolonged periods of time on cytotoxic T cells to be effective. In our experiments, we studied redirected lysis for 24 or 72 hours and T cell proliferation for 120 hours. Pre-existing toxins in secretory granules of effector T cells and their discharge during synapse formation may be very difficult to intercept, for instance, by mechanisms that work in T cells by transcriptional repression (like TGF-β) or by inhibition of T cell proliferation (like IDO).

In our experiments, we targeted CHO transfectants and tumor cells with high to moderate EpCAM expression. The expression of tumor antigen on tumor cells can be very heterogeneous. It is possible, that only a small number of target antigens per cell could result in reduced t cell activation, leaving the t cells more susceptible to inhibitory signals. Follow up studies will have to address whether t cell inhibition increases with decreasing levels of target antigens.

The BiTE^®^ principle is not dependent on the generation, expansion, epitope spreading and migration of specific T cell clones. This in itself may circumvent a number of evasion mechanisms that impact the latter.

How can the dose of AMG 110 eventually compensate for inhibitory effects of evasion factors? AMG 110 was shown to induce formation of a normally structured, functional cytolytic synapse between T cells and EpCAM-expressing target cells [33]. It is conceivable that by increasing the concentration of AMG 110, this BiTE^®^-induced synapse is getting enlarged, which may allow for delivery of more granzymes and perforin across the synapse.

Future studies need to investigate whether the surface of BiTE^®^-induced synapses is scalable and this way capable of delivering various doses of toxins in relationship to the BiTE^®^ concentration.

Even if certain evasion mechanisms can impact BiTE^®^-engaged T cells in their proliferation or effector function, as here observed for IDO, PD-L1, serpin PI-9 or TGF-β, there may be means to enhance a reduced BiTE^®^ function by pharmacological intervention. We here show that antibodies neutralizing the PD-1/PD-L1 axis or TGF-β can relieve an inhibition of redirected lysis. Likewise, inhibitors of IDO may be able to restore BiTE^®^-induced T cell cycling. With the aggressive clinical development of antibodies blocking the PD-1/PD-L1 axis and other checkpoint inhibitors, pharmacological tools will become available to clinically investigate whether BiTE^®^ antibody constructs can profit from a blockade of immune evasion.

## Supporting Information

S1 Fig Expression of escape proteins in CHO EpCAM transfectants and six human tumor cell lines. (A) Intracellular FACS analysis of PI-9 expression in fixed and permeabilized tumor cell lines and CHO EpCAM PI-9 transfectants. For comparison of expression levels the relative median (= median sample/median control) of the samples were compared with reference to the CHO EpCAM PI-9 cells. (B) FACS analysis of PD-L1 expression with unlabeled cells (open), untreated (black) and IFNγ pretreated (grey) cell lines labeled with anti-human PD-L1 antibody. For comparison of expression levels relative medians of all samples were calculated. (C) FACS analysis of EpCAM expression. For comparison of expression levels relative medians of all samples were calculated. (D) Western Blot Analysis of Bcl-2 expression and the corresponding loading control. 25 μg of total cell lysate were applied. Bcl-2 signals were analyzed with ImageJ software and evaluated with respect to loading control and CHO EpCAM Bcl-2 signal. (E) Western Blot analysis of IDO expression and the corresponding loading control. 100 μg protein of total cell lysate were applied. IDO signals were analyzed with ImageJ software and evaluated with respect to loading control and CHO EpCAM IDO signal. (F) ELISA analysis of IL-10 and (G) TGF-β expression in human cancer cell lines and CHO EpCAM tranfectants assessed in cell supernatant of 2,5x10^5^ /ml after 48 h of culturing. Error bars represent SEM out of the two assays. (H) FACS analysis of extracellular TGF-β expression in CHO EpCAM transfectants with unlabeled cells (grey), parental CHO cells (blue) and CHO EpCAM TGF-β transfectants (closed) labeled with anti-human TGF-β antibody.
(TIF)

S2 Fig Impact of rec. hum IL-10 and TGF-β on BiTE^®^-induced proliferation and target cell lysis. (A) Human CD3^+^ T cells were labeled with CFSE and co-cultured at effector to target (E:T) ratios of 1:8, 1:1 and 4:1 in 48-well plates in presence and absence of 1 μg/ml AMG 110 with CHO control cells, CHO EpCAM IL-10 cells or control cells in the presence of 10 ng/ml and 400 ng/ml hum IL-10 or with CHO control cells, CHO EpCAM TGF-β and control cells in the presence 100 ng/ml hum TGF-β. After 120 h, CFSE signals of CFSE-positive cells were analyzed using a FACS Canto^™^ II flow cytometer and FACS DIVA^™^ software. (B) Dose-dependent redirected target cell lysis of CHO EpCAM control cells, CHO EpCAM-IL10 transfectans and control cells in presence of 10 ng/ml or 200 ng/ml hum IL-10 and dose-dependent redirected target cell lysis of CHO EpCAM control cells, CHO EpCAM TGF-β transfectans and control cells in presence of 80 ng/ml hum TGF-β. Percentage of target cell lysis was assessed by an FACS-based cytotoxicity assay after 72 h of co-culture with CD3^+^ T cells at an E:T ratio of 4:1 using a FACS Canto^™^ II flow cytometer. Mean EC_50_ values were calculated with GraphPad Prism software. Error bars represent SEM out of duplicates.
(TIF)

S3 Fig Statistical analysis of EC_50_ values and amplitudes of CHO escape transfectants and corresponding controls. (A) EC_50_ values and (B) amplitudes of all executed assays using CD8^+^ T cells as effector cell population were analyzed with the Grubb′s test to exclude significant outliers. P values were calculated using unpaired t tests with welch′s correction with a significance level * = p <0.05.
(TIF)

S4 Fig Impact of diverse immune escape mechanisms on target cell lysis by redirected CD3^+^ T cells. Dose-dependent redirected target cell lysis of CHO cell lines (A) stably transfected with one of six human evasions proteins and the target antigen human EpCAM compared to parental EpCAM^+^ CHO cells or parental EpCAM^+^ CHO cells in presence or absence of evasion protein Adenosine using CD3^+^ T cells as effector cell population. Percentage of target cell lysis was assessed by a FACS-based cytotoxicity assay after 72 h of co-culture with CD3^+^ T cells at an E:T ratio of 4:1 using a FACS Canto^™^ II flow cytometer. Mean EC_50_ values were calculated with GraphPad Prism software. Error bars represent SEM out of duplicates. For quantification of effects of immune escape mechanisms on BiTE^®^-mediated redirected target cell lysis. (B) Relative Change in EC_50_ and (C) relative change in amplitude were calculated as described in Fig 2. Error bars represent SEM out of the assays performed for each different cell line. The number of repetitions is indicated. Dose-dependent redirected target cell lysis of human tumor cell lines with and without inhibition of endogenous (D) PI9 expression by shRNA, neutralization of endogenous (E) TGF-β or (F) endogenous PD-L1 by addition of neutralizing antibodies after (D-E) 48 h and (F) 24 h.
(TIF)

S5 Fig PD-1 increases upon stimulation. FACS analysis of PD-1 expression in CD3^+^T cells that were cultured with/without CD3/CD28/IL-2 96h after isolation.
(TIF)

S6 Fig Adenosine decreases CD25 expression. FACS analysis of CD25 expression in CD3^+^T cells stimulated by CD3/CD28/IL-2 with/without 1 mM of Adenosine (ADO).
(TIF)

S7 Fig Functional analysis of rec. hum TGF-β, TGF-β from supernatant of CHO tranfectants and TGF-β neutralizing antibody. Intracellular FACS analysis of granzyme B (GrB) expression in CD3^+^ T cells (A) stimulated by CD3/CD28/IL-2 with/without 100 ng rec. hum TGF-β, (B) stimulated in CHO EpCAM control cell supernatant and (C) CHO EpCAM TGF-β supernatant +/- TGF-β neutralizing antibody.
(TIF)
