# Supplementary figures and images for: Impact of Diverse Immune Evasion Mechanisms of Cancer Cells on T Cells Engaged by EpCAM/CD3-Bispecific Antibody Construct AMG 110
Source: PLoS One. 2015 Oct 28;10(10):e0141669. doi: 10.1371/journal.pone.0141669 (PMC4624873; doi:10.1371/journal.pone.0141669)

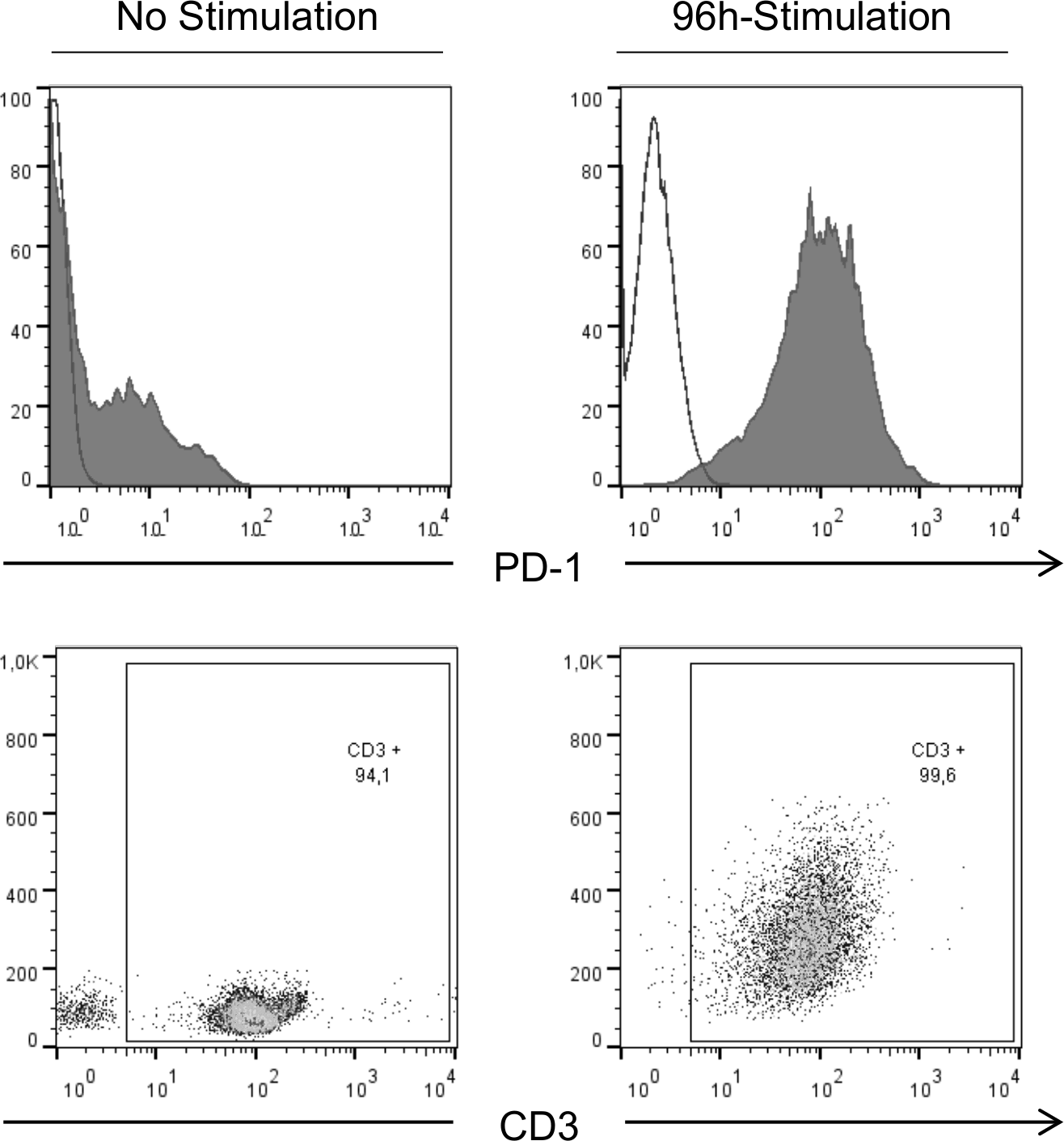

Supplement: S5 Fig — FACS analysis of PD-1 expression in CD3+T cells that were cultured with/without CD3/CD28/IL-2 96h after isolation. (TIF) [file pone.0141669.s005.tif]

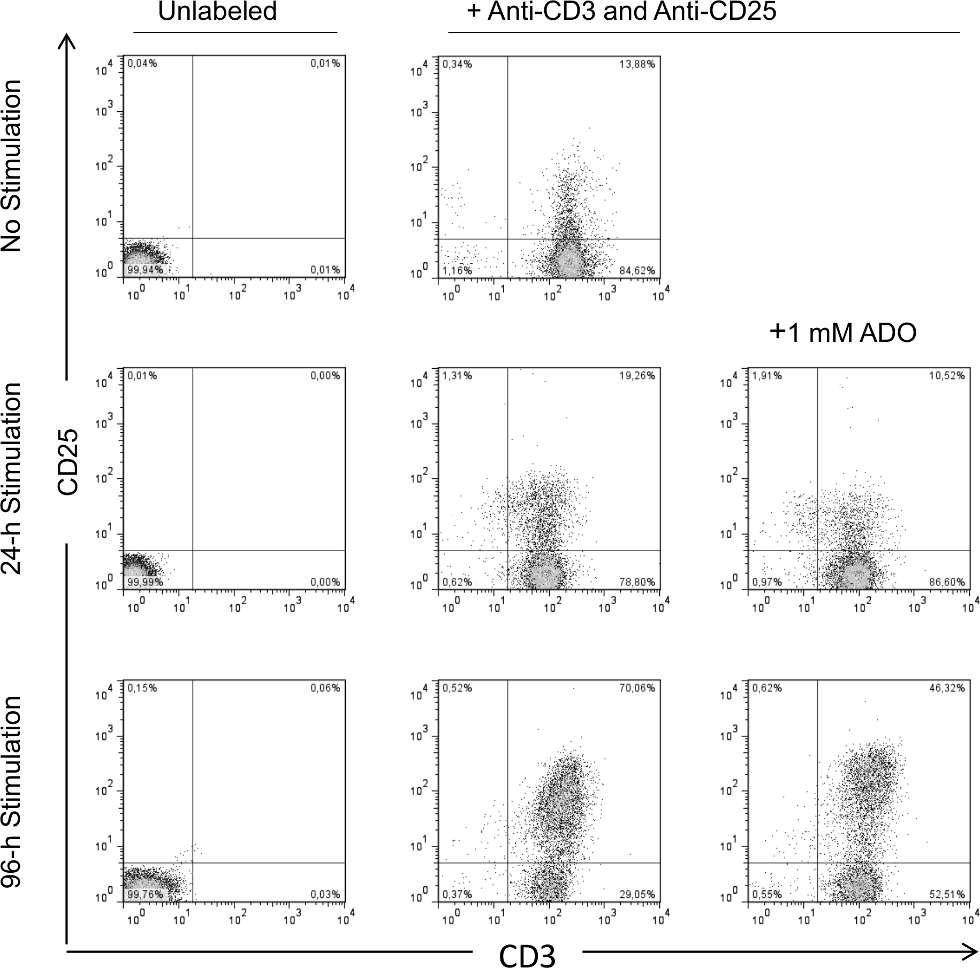

Supplement: S6 Fig — FACS analysis of CD25 expression in CD3+T cells stimulated by CD3/CD28/IL-2 with/without 1 mM of Adenosine (ADO). (TIF) [file pone.0141669.s006.tif]

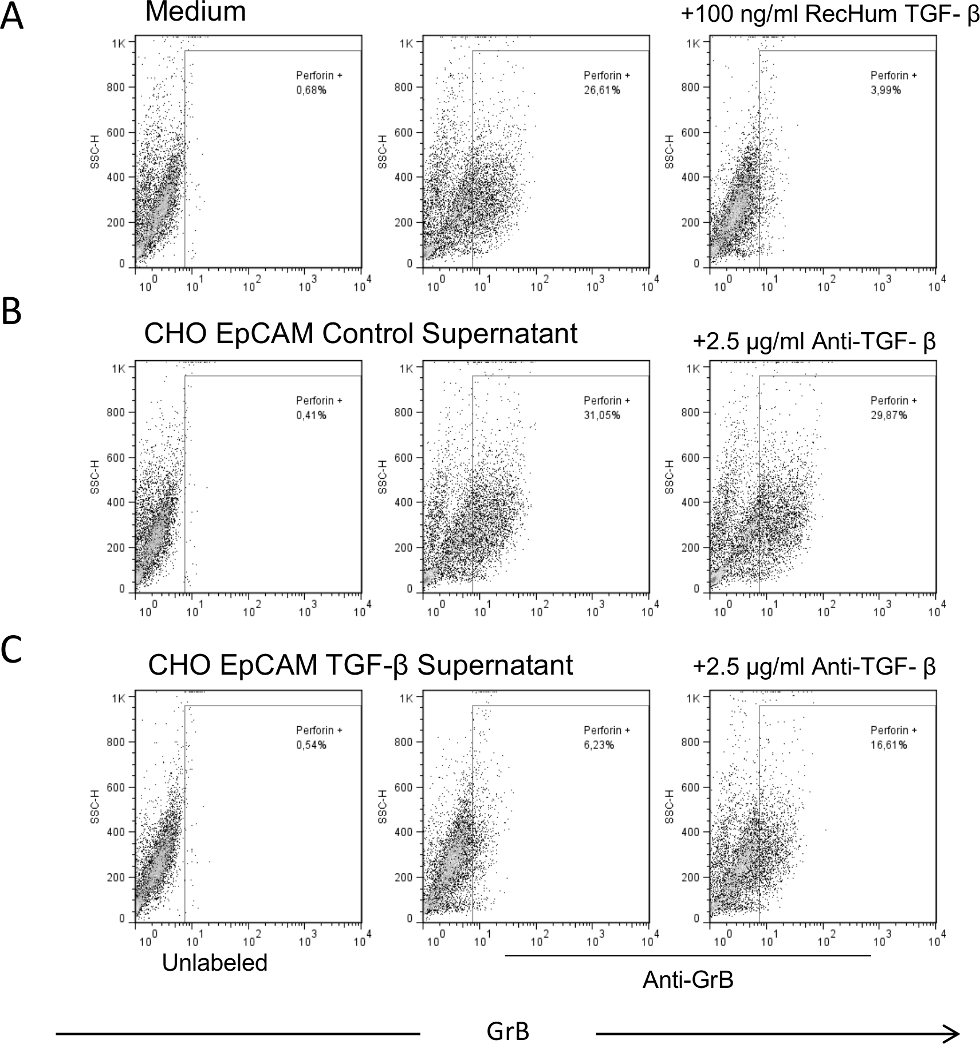

Supplement: S7 Fig — Intracellular FACS analysis of granzyme B (GrB) expression in CD3+ T cells (A) stimulated by CD3/CD28/IL-2 with/without 100 ng rec. hum TGF-β, (B) stimulated in CHO EpCAM control cell supernatant and (C) CHO EpCAM TGF-β supernatant +/- TGF-β neutralizing antibody. (TIF) [file pone.0141669.s007.tif]
